# Enteropeptidase: A Gene Associated with a Starvation Human Phenotype and a Novel Target for Obesity Treatment

**DOI:** 10.1371/journal.pone.0049612

**Published:** 2012-11-21

**Authors:** Sandrine Braud, Marco A. Ciufolini, Itzik Harosh

**Affiliations:** 1 ObeTherapy Biotechnology, Evry, France; 2 Department of Chemistry, The University of British Columbia, Vancouver, British Columbia, Canada; INSERM/UMR 1048, France

## Abstract

**Background:**

Obesity research focuses essentially on gene targets associated with the obese phenotype. None of these targets have yet provided a viable drug therapy. Focusing instead on genes that are involved in energy absorption and that are associated with a “human starvation phenotype”, we have identified enteropeptidase (EP), a gene associated with congenital enteropeptidase deficiency, as a novel target for obesity treatment. The advantages of this target are that the gene is expressed exclusively in the brush border of the intestine; it is peripheral and not redundant.

**Methodology/Principal Findings:**

Potent and selective EP inhibitors were designed around a boroarginine or borolysine motif. Oral administration of these compounds to mice restricted the bioavailability of dietary energy, and in a long-term treatment it significantly diminished the rate of increase in body weight, despite *ad libitum* food intake. No adverse reactions of the type seen with lipase inhibitors, such as diarrhea or steatorrhea, were observed. This validates EP as a novel, druggable target for obesity treatment.

**Conclusions:**

*In vivo* testing of novel boroarginine or borolysine-based EP inhibitors validates a novel approach to the treatment of obesity.

## Introduction

Obesity is a complex metabolic disorder in which many environmental factors and numerous genes are implicated [1], [2]. Past research aiming to develop drug treatments for obesity and type II diabetes has targeted genes that are associated with an obese human phenotype. Indeed, considerable effort has been devoted to developing drugs against these so called “obesity genes,” all of which are involved, directly or indirectly, in energy management; e.g., control of appetite, satiety, or thermogenesis; fatty acid, carbohydrate and protein metabolisms; energy generation; etc. [3], [4]. In humans, however, obesity is rarely attributable to the function of a single gene (wild or mutated). Moreover, the high redundancy of genes involved in energy management makes it unlikely that obesity will ever be controlled by targeting just one gene – *unless such a gene target is associated with a lean or starvation human phenotype*.

We posited that if a monogenic slimness disease, or a phenotype displaying a deficiency in energy absorption, could be identified, then the implicated gene would be likely to play a critical role in the lean phenotype. If this gene effect is not compensated by other mechanisms, then it could be a potential target for new anti-obesity drugs [5]. Such a gene might also be a more judicious target than the obesity genes in obese patients.

**Figure 1:**
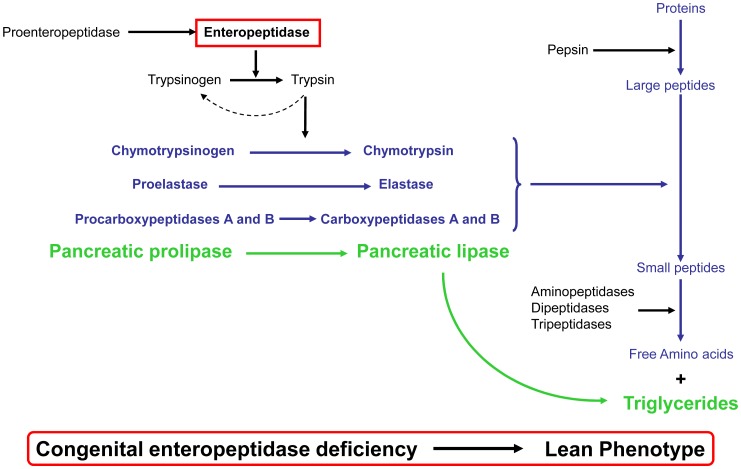
Cascade of biochemical events starting with proenteropeptidase. Enteropeptidase converts inactive trypsinogen into active trypsin, which in turn converts the other pancreatic zymogens, chymotrypsinogen, proelastase, carboxypeptidases A and B, and prolipase, into active enzymes.

Among the known genetic diseases associated with starvation human phenotype, *congenital enteropeptidase deficiency* (CEP, OMIM#226200) attracted our attention. Enteropeptidase (EP, also termed enterokinase) is a serine protease (EC 3.4.21.9) localized on the brush border of the duodenum. This enzyme catalyzes the conversion of inactive trypsinogen into active trypsin *via* the cleavage of the acidic propeptide from trypsinogen [6]. In turn, the activation of trypsin initiates a cascade of proteolytic reactions leading to the activation of many pancreatic zymogens, such as chymotrypsinogen, proelastase, procarboxypeptidases A and B, and prolipase, allowing absorption of amino acids and triglycerides (Fig. 1) [7].

Congenital EP deficiency is an extremely rare, recessive inherited disorder (so far only 13 cases have been reported in the scientific literature [8]) that results in failure to thrive, diarrhea, anemia, hypoproteinemia and edema [9], [10], [11], [12]. This condition is usually successfully treated by pancreatic enzyme replacement or by dietary administration of protein hydrolysate [10], [11], [12], [13], [14]. *In vitro* studies of small intestinal mucosal biopsy specimens suggest that EP deficiency is due to the formation of structurally altered enzymes with no EP activity [15].

The foregoing suggests that EP activity may serve as selective and efficient target for treating metabolic disorders. Whereas complete inhibition of EP would cause the undesirable effects seen in patients affected by CEP, partial inhibition should diminish the efficiency of energy absorption via the gastrointestinal (GI) tract. A 15–20% reduction in the daily absorption of energy deriving from both proteins and fatty acids should have a significant impact on long-term weight management, and it should be a more effective weight-control measure than a treatment based only on pancreatic lipase inhibitors such as Xenical or Alli. It should be noted that these drugs promote the accumulation of undigested lipids in the intestinal tract, resulting in leaky stool and diarrhea. An added advantage of partial EP inhibition is that the mixture of undigested lipids and proteins would be more consistent than just fat, arguably diminishing or suppressing the above unpleasant effects.

## Results and Discussion

A drug discovery program was launched on the basis of available structural [16], [17] and biochemical information. Enteropeptidase is known to be highly specific for the sequence (Asp)_4_-Lys-Ile of its trypsinogen substrate, and it acts by cleaving the Lys-Ile bond [7]. First, the selectivity of EP for particular amino acid sequences was evaluated by the use of commercial peptide substrates and inhibitors of serine proteases. This revealed a marked preference for specific amino acid arrangements. Rational drug design subsequently led to the identification of potent and selective enteropeptidase inhibitors based on a tripeptide motif.

Relative to such first-generation inhibitors, a noticeable improvement in both activity and selectivity was achieved by replacing the C-terminal amino acid with the corresponding aminoboronic acid. The boron atom in the resulting pseudopeptides is believed to interact strongly with a serine residue, which is part of the catalytic triad of EP (formed by His^57^, Asp^102^ and Ser^195^) [17]. It should be noted that boronic acid-based pseudopeptides constitute an emerging class of exciting, successful and safe drugs for the treatment of various pathologies [18], [19], [20]. An especially significant example is the proteasome inhibitor, Bortezomid (Velcade®) [21].

**Figure 2:**
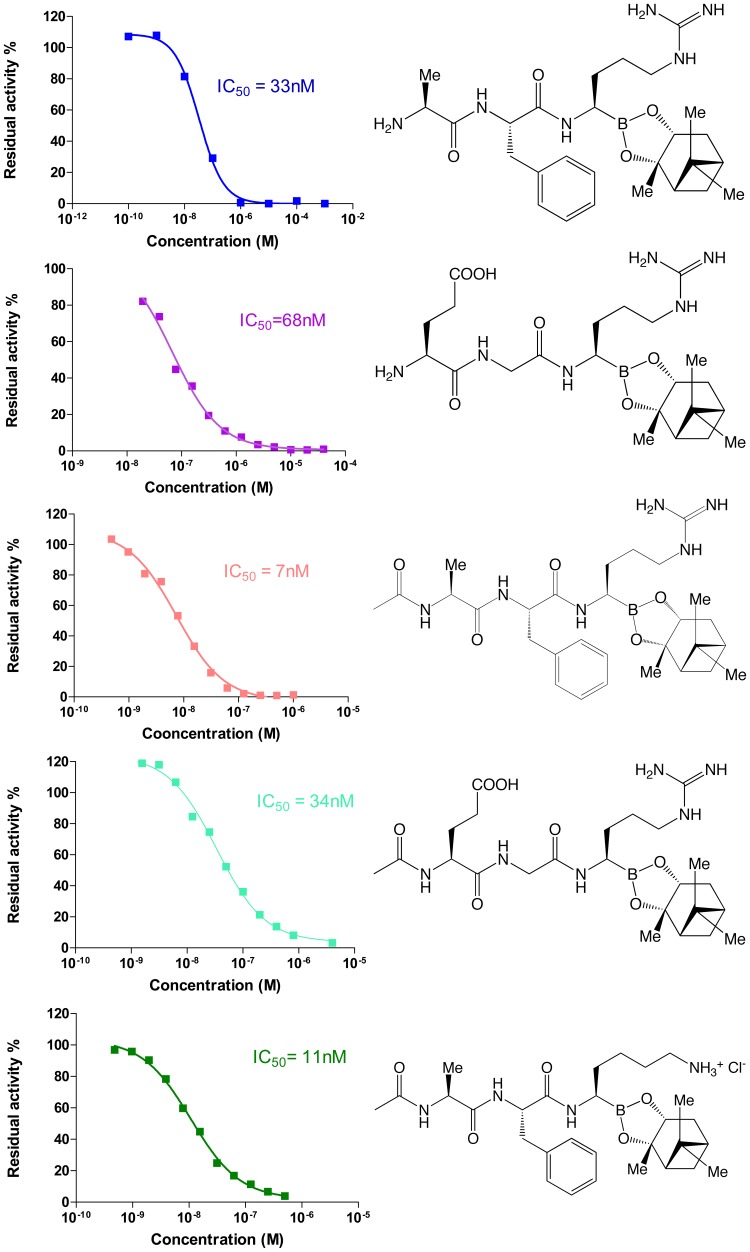
Inhibition of enteropeptidase by representative boropeptides. Enteropeptidase was incubated with increasing concentrations of inhibitor and residual activity was measured in presence of a colorimetric substrate at 420 nm during 1 hour to determine IC_50_.

Representative borotripeptides developed in the course of these studies are shown in Figure 2. These optimized, drug-like substances displayed *in vitro* IC_50_’s against EP ranging from 7 to 68 nM (Fig. 2), and were quite stable in gastric fluid (*in vitro*) at pH 1.2 and in the presence of pepsin for 20 hours (not shown). More importantly, they displayed excellent selectivity relative to other enzyme families (serine and aspartate proteases, metalloproteases, lipase, glycosidase). Indeed, the boropeptide compounds had no inhibitory effect on chymotrypsin, elastase, carboxypeptidase A1, carboxypeptidase B1, pancreatic lipase, DPPIV, and amylase, while they displayed very weak activity against some serine proteases sharing similarities with EP [22]. Such off-target activities, however, are of little concern thanks to the favorable pharmacokinetic properties of the inhibitors (*vide infra*). Selectivity in the desired sense was achieved by the judicious positioning of preferred amino acids at key positions. Considering the IC_50_’s against EP and the ease of chemical synthesis of OBE-2008 (incorporating a borolysine) compared to the 4 other borotripeptides (bearing a boroarginine), OBE-2008 was chosen for this study (see Supporting Information).

**Figure 3:**
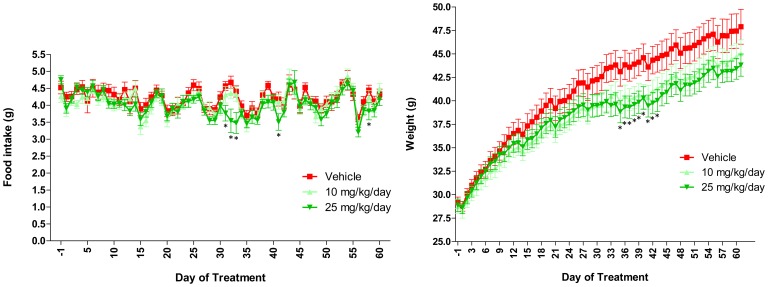
Effect of OBE-2008 administered before night phase on food consumption (left) and body weight (right) in mice. Red = control; dark green = dose of 10 mg/kg/day; light green = dose of 25 mg/kg/day. Number of mice per group n = 10. Statistical comparison between control and treated group were conducted by Dunnett’s tests. Asterisks (*) indicate p values versus control. A p value of<0.05 was considered to show a statistically significant difference.

To validate EP as a target for the treatment of obesity *in vivo*, the weight-reducing effectiveness of OBE-2008 was evaluated in a mouse model of diet-induced obesity (DIO). As seen in Figure 3, OBE-2008 had no effect on food consumption, but in a long-term treatment (up to 9 weeks) at daily doses of 10 and 25 mg/kg, it reduced weight gain by 6 and 10%, respectively, relative to a control group, in spite of *ad libitum* daily food intake. Unlike lipase inhibitors, OBE-2008 produced no side effects such as steatorrhea or diarrhea. As indicated earlier, this is attributable to the fact that OBE-2008 inhibits both protein and fat absorption. Therefore, the undigested intestinal content (a mixture of protein and lipids) is of greater consistency, resulting in a more solid, less oily stool.

**Figure 4:**
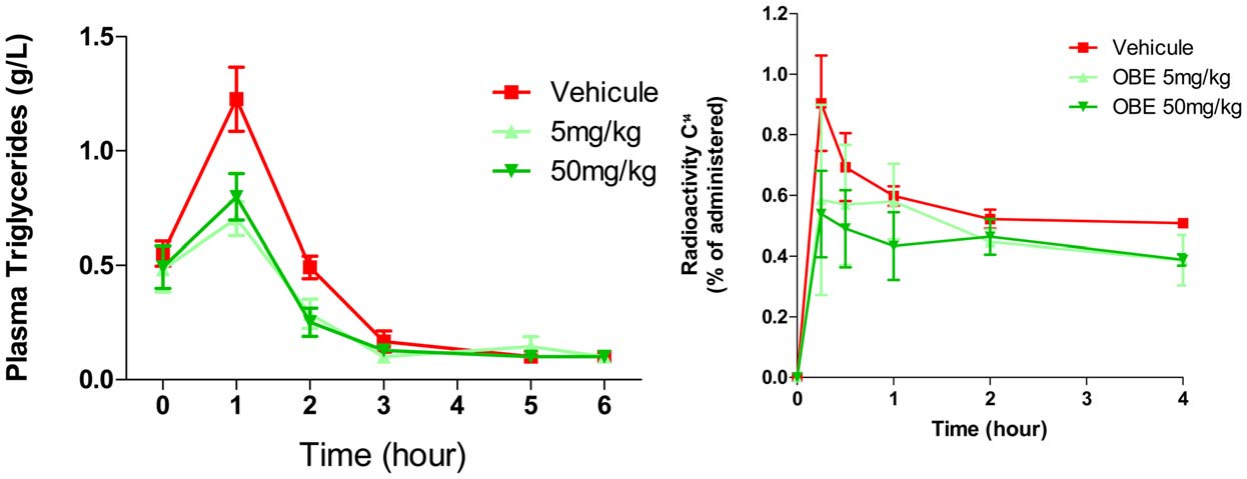
The mechanism-based efficacy of OBE-2008 on enteropeptidase was evaluated based on triglycerides (left) and amino acids (right) absorption. Red = control; dark green = dose of 5 mg/kg; light green = dose of 50 mg/kg. Number of mice per group n = 3 **(right)** for amino acids, and n = 8 **(left)** for triglycerides. Statistical comparison between control and treated group were conducted by two-sided unpaired Student’s tests. Asterisks (*) indicate p values versus control. A p value of<0.05 was considered to show a statistically significant difference.

Figure 4 illustrates the effect of OBE-2008 on fat and protein absorption in fasted mice pre-treated with OBE-2008 at doses of 5 mg/kg and 50 mg/kg, respectively, vs. untreated fasted mice. All animals were fed a mixture of fatty acids. In the two treated groups, the maximum level of triglycerides in plasma, after 1 hour, was substantially lower than in the control group, and it was followed by a rapid decrease in concentration up to 6 hours post-dosing. As estimated by computing the area under the curve (AUC), OBE-2008 significantly decreased (p value<0.05) fatty acids absorption by 33% and 32% at doses of 5 mg/kg and 50 mg/kg, respectively. The effect of OBE-2008 on protein absorption was evaluated in a similar fashion. Fasted mice pre-treated with OBE-2008 prior to gavage with a solution of radiolabeled proteins experienced a marked decrease in protein absorption during 240 minutes of follow-up monitoring. Thus total protein absorption assessed by AUC decreased by 17% and 22% in mice dosed with 5 mg/kg and 50 mg/kg of OBE-2008, respectively. However due to the restricted number of animal and variability, those diminution did not reach a statistically significant level. Altogether, the observed inhibitory effects on triglycerides and amino acids absorption support a mechanism of action based on enteropeptidase inhibition by OBE-2008 leading to reduction of calories absorption.

**Figure 5:**
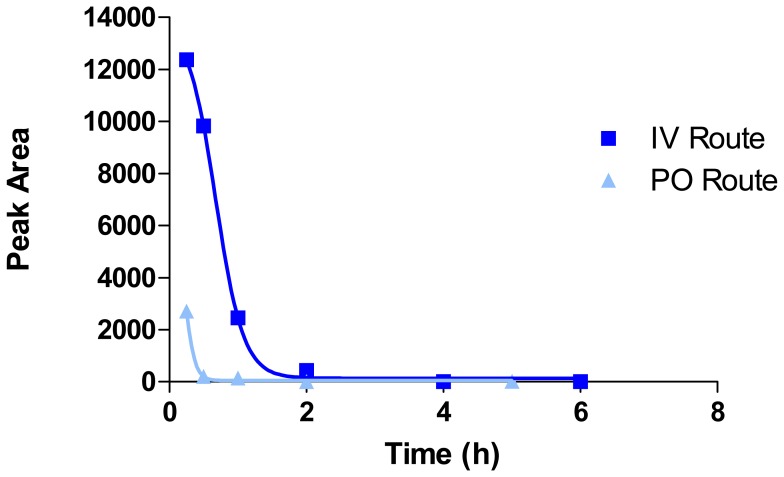
Pharmacokinetics of OBE-2008 in mice following intravenous (1 **mg/kg, dark blue) and** ***oral*** **(10 mg/kg, light blue) dosing.** Number of mice per group n = 3.

The pharmacokinetics of OBE-2008 in mice was investigated by comparing intravenous (1 mg/kg) and oral (10 mg/kg) administrations. Following intravenous injection, OBE-2008 was rapidly eliminated from the plasma with a half-life of minutes, and it was undetectable after 2 hours. Negligible absorption occurred after oral administration, with a calculated bioavailability of 2–3% (Fig. 5). Due to negligible absorption, fast elimination and rapid degradation in plasma, systemic exposure to OBE-2008 can be considered to be negligible after oral administration. Therefore, the weak off-target activity alluded-to earlier is of little or no concern, and few – if any – side effects are anticipated.

The results of our animal studies suggest that partial inhibition of EP through chemotherapeutic intervention, as described herein, is unlikely to lead to the nefarious effects observed in individuals affected by congenital EP deficiency. Instead, it will produce a limited degree of malabsorption, in the same way as bariatric surgery does, but without the notorious side effects, risks, and costs associated with surgical intervention [23]. Moreover, and in contrast to surgery, the extent of malabsorption induced by EP inhibitors can be tailored to individual patients by adjusting the dose of medication. Judicious dosing is also likely to alleviate or eliminate side effects such as steatorrhea/diarrhea (recall, no such effects were observed in our animal study) often seen in human patients treated with lipase inhibitors such as Orlistat (the only currently available drug for the treatment of obesity, recently commercialized over-the-counter under the brand name Alli). More recently, two new drugs were approved by the FDA: Lorcaserin, which is a selective 5-HT 2C receptor agonist [24], and qsymia which is a combination of phenetermine and topiramate [25]. Both new drugs are centrally acting appetite suppressors and increase the feeling of fullness. By contrast, OBE-2008 does not affect appetite (Fig. 3 left) and acts peripherally by inhibiting energy absorption deriving from proteins and fatty acids. This is in concordance with the phenotype of the enteropeptidase gene as described [8]. The daily requirement of protein is estimated to be between 40–70 grams per day depending on gender, age and activity. However, most experts agree that most people consume twice as much protein as they need.

A study in which strength athletes were compared with sedentary individuals (3 randomly designed groups on a low (0.86 g/kg), moderate (1.40 g/kg) and high (2.40 g/kg) protein diet) found that varying protein intake had no effect on lean body mass indexes of when creatinine excretion and body density was measured for either group [26]. This however, gives a relative large window to inhibit protein absorption since an average protein consumption is around 150 grams/day taking into consideration that an average steak is 200 grams and that protein can be found also in eggs, cheese, milk etc. It should always be kept in mind that excess proteins is transformed into fatty acids and stored in adipose tissues. Therefore reducing energy absorption deriving from both proteins and fatty acids should result in weight loss, and it should be more efficient than blocking only fatty acids absorption, e.g. with Orlistat. Furthermore, inhibition of fat absorption should also have a beneficial effect on lipid metabolism and cardiovascular health. Indeed, it has been shown that reducing fat absorption with Xenical has a direct effect on lipid profile in the blood and a beneficial long term effect on the cardiovascular system [27], [28].

In summary, a new approach to the treatment of obesity and associated conditions targets genes associated with a “starvation human phenotype”. This has led to the identification of enteropeptidase as a potential target for anti-obesity agents. *In vivo* inhibition of enteropeptidase with boropeptides has had a direct effect on weight management, due to inhibition of fatty acid and protein absorptions. Enteropeptidase is thus a good target for obesity treatment. Inhibition of EP activity so as to reduce daily absorption of energy by 15–20% should have a significant effect on weight management in the long term and may provide a selective and efficient approach to the treatment of obesity. We remain confident that anti-obesity agents that target genes associated with a “lean human phenotype” greatly increase the probability of successful treatment, as compared to those that target genes associated with an obese phenotype.

## Materials and Methods

### Enteropeptidase *in vitro* Test

A mix of enteropeptidase at 58.8 nM (final) and thermolysine at 1.58 ng/l (final) in TCN buffer was prepared and incubated at 37°C during 30 minutes for activation. Phosphoramidon (10 µM final) was added to stop the activation by thermolysine. In 17 µl of TCN, 1 µl of active enzyme (2.9 nM final) and 2 µl Np Tosyl Gly Pro Arg pNa (1 mM final) were mixed, just before reading. The absorbance was measured at 405 nm on EnVision (Perkin Elmer). In 15 µl of TCN, 1 µl of active enzyme and 2 µl of OBE lead at different concentrations were mixed, and incubated at RT (room temperature) for 30 minutes; 2 µl Np Tosyl Gly Pro Arg pNa (1 mM final) was added just before reading. The absorbance was measured at 405 nm on EnVision (Perkin Elmer).

### Long Term Effect of OBE Lead Compound on Mice Fed with DIO Regimen

The anti-obesity effect of OBE lead in a diet induced obesity model (45% kcal from lipids and 20% from proteins, reference 824053 from Special Diet Services, UK) was evaluated following daily oral administration in mice for 63 days. The study involved 3 groups of 10 male Swiss mice; Group 1: water; Group 2: OBE lead at 10 mg/kg/day; Group 3: OBE lead at 25 mg/kg/day. Animal were weighted daily. Food consumption was measured daily for each animal, except during fasted periods. For all groups, the food was given *ad libitum.* Body weights are given as means ± SEM (Standard Error of the Mean). The effects of test items on body weight and food consumption were expressed as absolute values and compared with those of the vehicle using multiple comparison procedure with a Dunnett’s test in case of significance (P≤0.05).

### Effect of OBE Lead Compound on Triglycerides Absorption in Mouse

The aim of the study was to evaluate the duration of effect of OBE lead on triglycerides absorption in mice after oral administration. The study involved 3 groups of 8 male animals each. Group 1: water; Group 2: OBE lead at 5 mg/kg; Group 3: OBE lead at 50 mg/kg. On the day of the test, all animals were given an oral fat overload. The preparation to be administered (OBE lead or its vehicle) was diluted at 1/100 in 10 mL of clinoleic acid 20. In all animals, about 60 µL were sampled via retro orbital sinus at time points: before dosing, 1, 2, 3, 5 and 6 hours post-dosing. The blood samples were prepared within 30 minutes of sampling by centrifugation for 10 minutes at 1500 g. All plasma samples were dosed for triglycerides. The effects of test items on triglycerides absorption were conducted by two-side unpaired Student’s test. A p value of<0.05 was considered to show a statistically significant difference.

### Effect of OBE Lead on Protein Absorption in Mouse

The aim of the study was to evaluate the duration of effect of OBE lead on absorption of ^14^C-proteins after oral administration in mice. Fifteen male Swiss mice were divided in 3 groups; Group 1: water and ^14^C-proteins; Group 2: OBE lead 5 mg/Kg and ^14^C-proteins; Group 3: OBE lead 50 mg/Kg and ^14^C-proteins. A preparation containing 5 ml of Clinoleic 20 (Baxter Ref DDB9500) with 1 g of glucose (Sigma; ref:G8270) and 1 g of casein (Sigma; ref: C3400) was prepared. The radioactive solution containing the ^14^C proteins (GE Healthcare; ref: CFA626) was concentrated in order to reach a concentration of ca 3.3 µCi/mL. The radioactive preparation (ca 33.3 µCi/kg) and OBE lead were orally administered by intragastric gavage. Plasma aliquots were removed at time points 15, 30, 60, 120 and 240 minutes. About 4 ml *Pico-fluor 40* was added and after shaking, the total radioactivity contained within the different samples was determined by liquid scintillation using a Packard 1900 CA spectrometer equipped with an external standard system. A quenching curve for calibration purposes was set up using a ^14^C quenched set supplied by Packard Instruments. Liquid scintillation (LS) counting was carried out with 2 sigma = 2%, and for a maximum duration of 5 minutes (according to the method created in the 1900CA® software). The effects of test items on proteins absorption were conducted by two-side unpaired Student’s test. A p value of<0.05 was considered to show a statistically significant difference.

### Pharmacokinetics of OBE Lead Compound

This study aimed to determine the fate of OBE lead in mice after intravenous (IV; 1 mg/kg) and oral (PO; 10 mg/kg) administration. Blood was taken from two animals at each sampling time from T = 0 to T = 6 h after IV administration, and from T = 0 to T = 24 h after oral administration. Samples were analyzed for OBE lead using LC/MS/MS methodology. For each IV and PO routes, three male Swiss mice aged 10-weeks were included in the experiment. The animals OBE IV-1, OBE IV-2 and OBE IV-3 were treated at T = 0 with 1 mg/kg of OBE lead using 2 mL/kg. The injection was performed in the jugular vein under isoflurane anaesthesia. The animals OBE PO-1, OBE PO-2 and OBE PO-3 were treated at T = 0 with 10 mg/kg of OBE lead using 5 mL/kg. The solution to be administered was delivered by gavage, using a gastric cannula. About 50–75 µL of blood were sampled from the retro orbital sinus. The blood was immediately centrifuged at 3500 rpm (+4°C) for 5 minutes. Protein precipitation was conducted by adding a volume of 400 µL of acetonitrile to 10 µL of plasma in Teflon vials. After centrifugation, an aliquot of the supernatant (+4°C auto-sampler; injected volume = 10 µL) was immediately analyzed by LC/MS/MS (column: HILIC Silica 5 µM 2.1×150 mm; isocratic elution using an ACN/pH3 buffer (70/30, V/V) eluant containing 1% formic acid; flow rate = 0.25 mL/min. The MS system operated in the ESI mode, and the ionization source was heated at 350°C). Chromatographic peaks areas were than calculated and plotted against time.

## Supporting Information

Protocol S1 **Preparation of representative borotripeptide inhibitors: synthesis of OBE 2001 and OBE 2008.**
(DOCX)
