# Supplementary material for: Enteropeptidase: A Gene Associated with a Starvation Human Phenotype and a Novel Target for Obesity Treatment
Source: PLoS One. 2012 Nov 21;7(11):e49612. doi: 10.1371/journal.pone.0049612 (PMC3504148; doi:10.1371/journal.pone.0049612)
Supplement: Protocol S1 — Preparation of representative borotripeptide inhibitors: synthesis of OBE 2001 and OBE 2008. (DOCX) [file pone.0049612.s001.docx]

**Enteropeptidase: a gene associated with a starvation human phenotype and a novel target for obesity treatment**

**Sandrine Braud^1^, Marco A. Ciufolini^2^ and Itzik Harosh^1*^**

**1** ObeTherapy Biotechnology, 4 rue Pierre Fontaine, 91058 Evry, France. **2** The University of British Columbia, Department of Chemistry, 2036 Main Mall, Vancouver B.C., V6T 1Z1, Canada

**Protocol S1**

**Preparation of representative borotripeptide inhibitors: synthesis of OBE 2001 and OBE 2008**

**A. General synthetic scheme**

**B. Representative experimental procedures: preparation of AcNH-Ala-Phe-BoroArg**

Compounds **2a**-**6a** are known and were prepared by the route described in the literature (Lebarbier, C.; Carreaux, B.; Boucher, J. L. *Bioorg. Med. Chem. Lett.* **1998**, *8*, 2573). The same procedure was employed for the formation of compounds **2b**-**6b**. All reactions were performed under dry argon in flame- or oven-dried flasks equipped with Teflon™ stirbars. All flasks were fitted with rubber septa for the introduction of substrates, reagents and solvents via syringe. All reagents and solvents were commercial products and used without further purification except THF (freshly distilled from Na/benzophenone under Ar) and CH_2_Cl_2_ (freshly distilled from CaH_2_ under Ar). Flash chromatography was performed on 230-400 mesh silica gel. Analytic TLC was carried out with Merck silica gel 60 plates with fluorescent indicator.

**Preparation of compound 3a**. Allyl bromide, **1a**, (15 g, 125 mmol) was added dropwise at 0 °C to catecholborane (15 g, 125 mmol ). After the completion of the addition, the reaction mixture was heated to 100 °C for 5 h, then it was cooled to 0 °C and a solution of pinanediol (21.3 g, 125 mmol) in THF (210 mL) was added dropwise. The reaction mixture was slowly allowed to warm up to room temperature and stirred overnight, then it was poured into 10% aqueous Na_2_CO_3_ solution. The mixture was extracted with ethyl acetate (2 x 200 mL). The combined extracts were washed with saturated brine solution, dried (Na_2_SO_4_), filtered and concentrated under vacuum. The residue was purified by column chromatography (pet ether /EtOAc, 95 : 5) to afford the known **3a** (16.9 g, 45%) as a pale yellow oil. The ^1^H NMR spectrum of this material was in accord with the one reported in the literature. ^1^H-NMR (300 MHz, CDCl_3_): *δ* 4.26 p.p.m. (dd, *J*_1_ = 10.2 Hz, *J*_2_ = 1.2 Hz, 1H), 3.43 (t, *J* = 8.1 Hz, 2H), 2.25 (m, 2H), 1.95 (m, 5H), 1.38 (s, 3H), 1.29 (s, 3H), 1.09 (d, *J* = 12.1 Hz, 1H), 0.96 (t, *J* = 9.3 Hz, 2H), 0.83 (s, 3H).

**Preparation of compound 4a**. Sodium azide (7.3 g, 112 mmol) was added portionwise at room temperature to a solution of **3a** (16.9 g, 56 mmol) in DMF (35 mL), and the resulting mixture was heated to 100 °C (oil bath temperature) for 60 min. The solution was then cooled to room temperature, poured into water and extracted with EtOAc (2 x 200 mL). The combined extracts were washed with saturated brine solution, dried (Na_2_SO_4_), filtered and concentrated under vacuum to furnish the known **4a** (12.5 g, 85 % ) as a pale yellow oil, which was used in the next step without purification. The ^1^H NMR spectrum of this material was in accord with the one reported in the literature. ^1^H-NMR (300 MHz, CDCl_3_): *δ* 4.26 p.p.m. (dd, *J*_1_ = 10.2 Hz, *J*_2_ = 2.1 Hz, 1H), 3.26 (t, *J* = 8.4 Hz, 2H), 2.21 (m, 2H), 2.04 (t, *J* = 6.3 Hz, 1H), 1.88 (m, 4H), 1.39 (s, 3H), 1.29 (s, 3H), 1.10 (d, *J* = 13.2 Hz, 1H), 0.86 (t, *J* = 9.0 Hz, 2H), 0.85 (s, 3H).

**Preparation of compound 5a**. Anhydrous THF (100 mL) and anhydrous CH_2_Cl_2_ (23 mL) was taken in a three neck flask under argon and was cooled to – 100 °C. A solution of n-BuLi (1.6 M in hexane, 35.7 ml, 57 mmol) was added dropwise very slowly on the side of the flask. After the completion of addition, the reaction mixture was maintained at –100 °C for 15 min, then a solution of **4a** (12.5 g, 47.5 mmol) in anhydrous THF (12.5 mL) was added dropwise. The mixture was warmed to –78 °C and stirred for 30 min. Anhydrous ZnCl_2_ (6.5 g, 47.5 mmol) was added rapidly. The mixture was stirred at – 78 °C for a further 15 min, then it was and allowed to warm up to room temperature. After stirring at room temperature for 2 h, the reaction mixture was poured into saturated aqueous NH_4_Cl solution and extracted with EtOAc (2 x 150 mL). The combined extracts were dried (Na_2_SO_4_), filtered and concentrated under vacuum. The residue of crude **5a** (11.8 g, 80%; known compound), an orange oil, was used in the next step without purification. The ^1^H NMR spectrum of this material was in accord with the one reported in the literature. ^1^H-NMR (300 MHz, CDCl_3_): *δ* 4.38 p.p.m. (dd, *J*_1_ = 10.2 Hz, *J*_2_ = 2.4 Hz, 1H), 3.49 (m, 1H), 3.30 (t, *J* = 7.8 Hz, 2H), 2.32 (m, 2H), 2.10 (t, *J* = 6.3 Hz, 1H), 1.93 (m, 6H), 1.43 (s, 3H), 1.30 (s, 3H), 1.15 (d, *J* = 13.2 Hz, 1H), 0.85 (s, 3H).

**Preparation of compound 6a**. A solution of KHMDS (0.5 M in toluene, 72 mL, 38 mmol) was added very slowly. to a cold (–78 °C), well stirred solution of crude **5a** (11.8 g, 38 mmol) in anhydrous THF (110 mL), under argon. The mixture was gradually allowed to reach room temperature and it was stirred at room temperature overnight. The solution was then concentrated under vacuum and the residual oil was triturated with hexane. The solid that separated was removed by filtration over Celite.^®^ The filtrate was cooled to – 78 °C and treated with a solution of 4M HCl in dioxane (10 mL), added dropwise under argon. The mixture was stirred for 15 min at – 78 °C, then it was gradually allowed to reach room temperature. After stirring at room temperature for 4.5 hrs, the solution was concentrated under vacuum. The residual white solid was suspended in hexane and stirred for 30 min at room temperature, then it was recovered by filtration and dried to furnish 4.3 g (35 %) of **6a** as a white solid. The ^1^H NMR spectrum of this material was in accord with the one reported in the literature. ^1^H-NMR (300 MHz, CDCl_3_): *δ* 8.35 p.p.m. (br s, 3H), 4.41 (dd, *J*_1_ = 10.5 Hz, *J*_2_ = 1.2 Hz, 1H), 3.34 (t, *J* = 7.8 Hz, 2H), 2.99 (br m, 1H), 2.06 (t, *J* = 6.3 Hz, 1H), 1.93 (m, 7H), 1.43 (s, 3H), 1.28 (s, 3H), 1.14 (d, *J* = 13.2 Hz, 1H), 0.83 (s, 3H).

**Preparation of compound compound 11 (OBE-2001)**. Compound **6a** (19.6 g, 59.7 mmol) was added to a cold (0 °C), well stirred solution of Ac-Ala-Phe-OH (16.6 g, 59.7 mmol), HBTU (27.2 g, 71.7 mmol) and *N*-methylmorpholine (23.4 mL, 239 mmol) in DMF (100 mL). The mixture was allowed to reach room temperature. After stirring overnight. the solution was poured into ice-water and extracted with chloroform (3 x 100 mL). The combined extracts were sequentially washed with aqueous 1M HCl, aqueous saturated NaHCO_3_, and brine; dried (Na_2_SO_4_), filtered, and concentrated under vacuum. This afforded **7a** (16.5 g, 50 %) as an off- white solid, which was pof sufficently good quality to be used without further purification. Thus, zinc dust (1.9 g, 29.9 mmol) was added portionwise at room temperature to a vigorously stirred solution of **7a** (16.5 g, 29.9 mmol) in ethanol (165 mL) containing suspended NH_4_Cl (3.3 g, 59.7 mmol). After stirring for 2 hrs, additional NH_4_Cl (3.3 g, 59.7 mmol) and zinc dust (1.9 g, 29.9 mmol) were added. After stirring for 2 more hrs at room temperature, the mixture was filtered over Celite.^®^ The solid residue was thoroughly washed with ethanol. The combined organic phases were cooled to 0 ° C and treated with 4 M HCl in dioxane (16.5 mL). The solution was stirred for 15 min, then it was concentrated under vacuum. The residue of crude **8** (9.3 g, 55 %), an off-white solid, was of sufficiently good quality to be used without further purification. Accordingly, a solution of **8** (1g, 1.8 mmol), 4-DMAP (435 mg, 3.6 mmol), and bis-BOC triflylguanidine (835 mg, 2.1 mmol) in 1,2-dichloroethane (10 mL) was heated at 60 °C for 30 min in a microwave reactor. The mixture was then cooled to room temperature and poured into chloroform (500 mL). The resulting solution was sequentially washed with aqueous 1 N HCl and saturated sodium chloride solution; dried (Na_2_SO_4_), filtered, and concentrated under vacuum to afford a residue of crude **10** (685 mg, 50 %) as an off-white solid. Commercial 4 M HCl in dioxane (15 mL) was added dropwise to a cold (0 °C) solution of **10** (6.3 g, 8.2 mmol) in anhydrous CH_2_Cl_2_ (50 mL). The reaction mixture was stirred overnight, during which time it was gradually allowed to warm to room temperature. The solution was evaporated to dryness *in vacuo* and the residue was purified by preparative HPLC to afford **11** (1.4 g, 30 %) as white solid of purity > 95%.

^1^H-NMR (250 MHz, MeOH-*d_4_*): *δ* 7.26 p.p.m. (m, 5H), 4.74 (t, *J* = 7.2 Hz, 1H), 4.24 (dd, *J*_1_ = 7.3 Hz, *J*_2_ = 0.9 Hz, 1H), 4.13 (q, *J* = 7.2 Hz, 1H), 3.15, (m, 4H), 2.55 (m, 1H), 2.37 (m, 1H), 2.16 (m, 1H), 1.96 (s, 3H), 1.84 (m, 3H), 1.54 (m, 4H), 1.40 (s, 3H), 1.30 (s, 3H), 0.91 (d, *J* = 7.2 Hz, 3H), 0.89 (s, 3H); ESI-MS (*m/z*): 569 [M+H]^+^.

**^1^H NMR Spectrum of Compound OBE2001**

**Preparation of compound 9 (OBE-2008)**. This substance (2.2 g) was prepared by the same procedure detailed above, but by starting with compound **1b**. The purity of crude product emerging from the Zn reduction step was assayed to be 85% (HPLC). Further purification by reverse phase HPLC determined a final purity of 99.3% (Atlantis d-C_18_ column, 75 x 4.6 mm, 5μm, 1.0 mL/min, MP-A - 0.1% TFA in water MP-B – MeCN)

^1^H-NMR (300 MHz, MeOH-*d_4_*): *δ* 8.80 ppm (br s, 1H), 8.84 (m, 2H), 7.67 (br s, 3H), 7.23 (m, 5H), 4.54 (m, 1H), 4.12 (m, 3H), 2.93 (m, 2H), 2.72 (m, 2H), 2.41 (m, 1H), 2.22, (m, 1H), 2.04 (m, 1H), 1.85 (m, 1H), 1.82 (s, 3H), 1.65 (m, 1H), 1.48-1.15 (m, 6H), 1.27 (s, 3H), 1.23 (s, 3H), 0.91 (d, *J* = 7.2 Hz, 3H), 0.82 (s, 3H); ESI-MS (*m/z*): 541 [M+H]^+^

**^1^H NMR Spectrum of Compound OBE2008**
